# Right ventricular dysfunction in structural tricuspid interventions

**DOI:** 10.1093/ehjimp/qyaf135

**Published:** 2025-10-29

**Authors:** Jonathan Lee, Eirini Beneki, Nikolaos Katsanakis, Edoardo Zancanaro, Monica Mukherjee, Edgar Argulian, Julia Grapsa

**Affiliations:** Department of Cardiology, Guys and St Thomas NHS Trust, London, United Kingdom; Department of Cardiology, CHUV Lausanne University Hospital, Lausanne, Switzerland; Department of Cardiology, Klinikum Dortmund gGmbH, Dortmund, Germany; Department of Cardiothoracic Surgery, Mainz Hospital, Mainz, Germany; Department of Cardiology, John Hopkins Hospital, Baltimore, MD, USA; Department of Cardiology, Mount Sinai Hospital, Icahn School of Medicine, New York, USA; Heart and Vascular Institute, Brigham and Women’s Hospital, Harvard Medical School, 15 Francis Street, Boston 02115, USA

**Keywords:** right ventricle, echocardiography, multimodality imaging, tricuspid interventions

## Abstract

With the growing global adoption of transcatheter tricuspid valve intervention (TTVI) and the increasing number of available devices, a comprehensive understanding of right heart dysfunction has become essential. Right heart dysfunction is frequently observed both before and after TTVI and is associated with adverse clinical outcomes. Therefore, a thorough understanding of right heart anatomy and physiology is critical for accurately assessing its pathological states. This review synthesizes current knowledge by integrating findings from major landmark studies on TTVI, with a focus on the available assessment tools for predicting patient outcomes. The anatomy section systematically reviews each component of the right heart—the right atrium, right ventricle, tricuspid valve, and pulmonary valve —while the physiology section emphasizes microstructural characteristics and the pressure-volume relationships. In addition, recommendations from the Tricuspid Valve Academic Research Consortium and the imaging parameters used in recent studies are discussed. Finally, future directions for imaging-based assessment of right heart function in the context of TTVI are highlighted.

## Introduction

Tricuspid structural interventions have grown significantly in the last 2 decades opening the pathways to explore strategies for best patient selection and outcomes. While initial trials employed suboptimal echocardiographic indices with multiple limitations, the need for more objective and reliable indices is greater than ever. This review focuses on a summary on right ventricular (RV) anatomy and physiology, an overview on how tricuspid and right heart indices have been employed in structural trials and the future perspectives on this topic.

## Anatomy of the right heart and tricuspid valve

### Anatomy of the right heart

The right atrium (RA) is divided into three main components: the smooth-walled venous portion, the trabeculated RA appendage and the anteroinferior vestibule transitioning to the tricuspid valve (TV). The smooth posterior wall receives the superior and inferior vena cava and the coronary sinus. The crista terminalis separates the smooth posterior wall from the trabeculated anterior region and is externally marked by the sulcus terminalis, a surface landmark for the sinoatrial node.^1,2^ Inferiorly, Koch's triangle, bounded by the tendon of Todaro, the coronary sinus ostium and the septal leaflet of the TV, houses the atrioventricular node, a crucial region in electrophysiological interventions.^2,3^

The RV is anatomically divided into three functional segments: the inflow tract, the trabeculated apical myocardium and the smooth-walled infundibulum leading to the pulmonary valve (PV).^1,2^ The crista supraventricularis separates inflow from outflow and extends into the parietal and septal bands.^4^ The septomarginal trabecula (moderator band), a prominent muscular structure, extends from the interventricular septum to the anterior papillary muscle, supporting part of the right bundle branch and providing coordinated electrical activation and mechanical contraction.^2,4^ The myocardial architecture of the RV is composed of superficial circumferential and deep longitudinal fibres, the latter contributing approximately 75% of systolic function under physiological conditions.^4,5^

### Anatomy and histology of the tricuspid valve

The TV is the largest valve and typically consists of three unequal leaflets: anterior, posterior and septal. However, anatomical variations such as bicuspid or quadricuspid configurations are commonly observed, even in normal populations.^6^ Posterior leaflet variation appears to be most common. The anterior leaflet is the largest and most mobile, while the septal leaflet adheres directly to the interventricular septum approximately 10 mm apically from the insertion of the anterior mitral leaflet, establishing the physiological atrioventricular offset. The valve is supported by a complex subvalvular apparatus involving the papillary muscles and chordae tendineae. The anterior papillary muscle provides chordae tendineae to the anterior and posterior leaflets, while the posterior papillary muscles support the posterior and septal leaflets. Septal papillary muscles are small and variable. Uniquely, chordae can originate directly from the septal wall, bypassing the discrete papillary muscles.^6,7^

Histologically, the TV leaflets are thinner than those of the mitral valve, tailored for low pressure haemodynamics. The supporting chordae are classified into several types, with ‘free edge’ and ‘deep’ chordae being characteristic of the TV.^7^ TV is notorious for dense and complex chordal apparatus. This should be taken into consideration when planning transcatheter TV intervention (TTVI), especially tricuspid edge-to-edge repair (TEER) procedures, since chordal complexity may impact the procedural outcome. The TV annulus (TA) is a dynamic, saddle-shaped structure composed predominantly of muscle and adipose tissue in the anterior and posterior segments, with minimal fibrous reinforcement along the septal side.^5–7^ Unlike the mitral annulus, the TV lacks a continuous fibrous skeleton, making it more compliant but more susceptible to dilatation, a process in which a 40% increase in annular size can cause significant regurgitation.^6^

### Anatomical relationship of the tricuspid and pulmonary valves with other structures

The anatomical relationship of the TV to adjacent structures has significant clinical implications. The right coronary artery (RCA) runs adjacent to the TA, particularly within 3 mm of its inferior segment, which poses a risk during transcatheter interventions. The atrioventricular node and His bundle are located close to the anteroseptal commissure, approximately 3–5 mm from the septal leaflet insertion.^6,7^ Histological differences between the sexes have also been reported: males have greater myocardial content and elastic fibres, whereas females have a larger annular diameter, even when adjusted for body surface area.^6^

Located anteriorly and superiorly to the aortic valve, the pulmonary valve consists of three semilunar cusps: anterior, right and left.^8,9^ The valve leaflets are thinner than their aortic counterparts, corresponding to the low pulmonary arterial pressures. Histologically, PV is composed of four layers—arterialis, fibrosa, spongiosa and ventricularis—which contribute to its mechanical strength and flexibility.^2,8^ The normal PV orifice area is approximately 3 cm², with congenital variations such as bicuspid or quadricuspid morphology being relatively rare but relevant to surgical planning.^9^

Blood supply to the right heart is predominantly from the RCA.^2^ Venous drainage is via the anterior cardiac veins and the small cardiac vein into the RA or coronary sinus.^10^ Autonomic innervation arises from the cardiac plexus, integrating sympathetic fibres from the thoracic ganglia and parasympathetic fibres from the vagus nerve, with intrinsic ganglia facilitating local reflex control.^10,11^

## Physiology of the right ventricle

### Myofibre architecture of the right ventricle

Appreciating the RV myoarchitecture is crucial to understand the function and contraction patterns of RV. The RV myofibres are arranged in two main layers. The subepicardial layer of cardiomyocytes is arranged circumferentially in a direction that is parallel to the atrioventricular groove. Towards the RV apex, the fibres turn slightly oblique and continue into the superficial layer of the left ventricular (LV) myocardium.^12^ The subendocardial layer of myocytes is longitudinally aligned (base to apex).^4^ The continuity between RV and LV muscle fibres, combined with shared myofibre tracts, the interventricular septum, and the pericardium, forms the basis of ventricular interdependence.^13^

### Mechanical aspects of ventricular contraction

RV contracts by three separate mechanisms: (1) contraction of the longitudinal fibres, which shortens along the long axis and draws the TA towards the apex; (2) contraction of the circumferential fibres with movement of the free wall towards the septum; and (3) traction on the free wall at the points of attachment to the LV secondary to LV contraction and LV-RV continuity of the superficial fibres.^12^

The RV contracts in a highly orchestrated fashion that occurs 20 to 50 miliseconds earlier in the inlet and trabeculated myocardium than the infundibulum, resulting in a peristalsis-like motion.^13,14^ Importantly, the RV has fewer oblique fibres than the LV, relying primarily on longitudinal contraction from the epicardium to the endocardium for forward flow^15^ and, unlike the LV, twisting and rotational movements contribute minimally. Flow studies showed less vortex forms in the RV, with a predominant helical flow towards the outflow tract, aided by the trabeculated apex.^16^ Additionally, LV contraction contributes to 20% to 40% of RV stroke volume and pulmonary flow in experimental models.^1,17,18^ The anatomic relationship between RV and LV is explained by shared septum, continuity of microfibre architecture and shared pericardial space explaining functional dependence of biventricular performance.

### RV haemodynamics

RV performance is influenced by pericardial constraint, ventricular interdependence, and heart rhythm.^19^ The quality of the RV is not in generating pressure, but rather in streamlining varying amounts of venous return into a relatively constant stroke volume (SV) that is ejected into the low-impedance pulmonary circulation with one-fourth of the LV stroke work.^13^ The large surface area compared to its volume, and a relatively thin wall, according to Laplace’s law, help the RV adapt to a broad spectrum of preload alterations except with brisk increments in afterload.^20^ Rapid changes in pre- or afterload lead to RV dilation in order to preserve SV. After several minutes, the heterometric adaptation is replaced by an homeometric adaptation with end-diastolic volume normalization and increased contractility.^4,20^

### Invasive haemodynamics

The intricate relationship between RV contractility, preload, and afterload can be more clearly understood through pressure-volume loop analysis. Pressure-volume analysis depicts instantaneous pressure-volume curves under different loading conditions and is considered the gold standard for characterizing ventricular function.^21^ End-systolic pressure-volume relationship is reasonably linear with a slope known as the end-systolic elastance, which is considered a load-independent index of RV contractility. RV diastolic function is largely determined by muscle mass, chamber geometry, diastolic muscle properties, and extracellular matrix mechanical properties. The slope of the line connecting the end-systolic pressure-volume point with the end-diastolic volume point on the volume axis represents the effective arterial elastance, a measure of total RV arterial load (including the impact of pulmonary vascular resistance and pulmonary capillary wedge pressure).^22^

### RV function in volume overload

Within physiological limits, an increase in RV volume improves myocardial contraction based on the Frank-Starling mechanism. Moreover, experimental studies demonstrate that RV contractility remains preserved in RV volume overload for long periods of time, although contractile reserve may be compromised.^23^ Beyond the physiological range, excessive RV volume loading can compress the LV and impair global ventricular function through ventricular interdependence. The primary mechanism is underfilling driven by septal displacement and changes in LV geometry, rather than reduced RV forward flow.^17^ An additional potential mechanism for impaired biventricular systolic performance is the reorientation of myocardial fibres, which could in theory interfere with myocardial mechanics.^24^

### RV function in pressure overload

The RV demonstrates a heightened sensitivity to changes in afterload compared to LV.^25^ In the context of chronic pressure overload, the RV initially responds with adaptive remodelling characterized by relatively preserved volumes and function along with concentric hypertrophy (increased mass to volume ratio hence decrease in wall tension).^26^ At this stage, functional status, exercise capacity, and cardiac output may remain relatively well preserved.

When the high pulmonary pressure persists or even further increases, the RV needs to undergo further remodelling, eventually leading to a maladaptive state, defined by RV dilation.^26,27^ Maladaptive remodelling is characterized by eccentric hypertrophy, progressive RV dilation, and mechanical dyssynchrony. Stroke volume is initially maintained through Frank-Starling mechanisms (heterometric adaptation); however, this compensatory response comes at the cost of elevated filling pressures. Over time, this leads to a decline in cardiac output and eventual clinical decompensation.^27^ As pulmonary hypertension progresses, RV shifts from predominantly longitudinal shortening to greater reliance on transverse wall motion. Impaired myocardial deformation and reduction in ejection are most pronounced at the apical segments.^28–30^

### Imaging endpoints and parameters in structural intervention studies

TTVI has emerged as a major focus of research in structural heart disease in recent years. While multiple devices are currently undergoing investigational evaluation, only a limited number have received regulatory approval. Although some devices have demonstrated improvements in quality of life, their impact on hard clinical endpoints remains to be conclusively established.^31–33^ To improve clinical outcomes in patients undergoing TTVI, a detailed understanding of post-procedural changes in cardiac structure and function is essential. Currently, therapeutic goals and definitions of clinically meaningful outcomes remain incompletely established. In this context, the standardization of imaging-based endpoints as surrogate markers represents a critical initial step towards elucidating the underlying disease mechanisms and the adaptive cardiac responses to intervention. Such standardized imaging metrics could facilitate more consistent assessment across studies, enable earlier identification of patients at risk for poor outcomes, and inform iterative refinement of device design and therapeutic strategies. Ultimately, this approach has the potential to translate into improved hard clinical endpoints, including survival and heart failure hospitalizations.

As efforts continue to identify the most accurate surrogate markers of RV performance and predictors of long-term clinical outcomes, it is important to summarize the current recommendations from the Tricuspid Valve Academic Research Consortium (TVARC)^34^ alongside imaging parameters assessed in studies on TTVI beyond the initial first-in-human experience.

### Defining procedural success

It has been proposed^34^ that an optimal procedural outcome following TTVI should be defined by achieving mild residual tricuspid regurgitation (TR) of (≤1+), with moderate (≤2+) TR considered acceptable, as residual TR of ≥ moderate severity has been consistently associated with adverse outcomess.^35^ The definition of procedural success should also include the absence of tricuspid stenosis (TV area ≧1.5 cm^2^, indexed tricuspid valve area ≧0.9 cm^2^/m^2^ (≧0.75 if body mass index >30 kg/m^2^), Doppler velocity index <2.2 and transtricuspid diastolic mean gradient (DMG) <5 mmHg. Since the threshold for intervention is severe or more TR (based on 5 grade classification), achieving a reduction to moderate (≤2+) or less has been universally adopted as a procedural success criterion across clinical studies.^32,36–41^ It has been used to define procedural success at different time points from baseline, 6 months and annually from 1 year through to 5 years. Some studies have additional or alternative requirements. In TRISCEND^37^ investigating the EVOQUE system (Edwards Lifesciences, Irvine, USA), the absence of clinically significant paravalvular leak was defined as procedural success. In PASTE^39^ investigating the PASCAL TEER device (Edwards Lifesciences, Irvine, USA), procedural success was defined as ≦moderate residual TR alongside transtricuspid DMG <5 mmHg and acute procedural success as a reduction of TR severity by at least 1 grade, the same as CLASP-TR^40^ also investigating the PASCAL device. In TRI-REPAIR^41^ investigating the Cardioband (Edwards Lifesciences, Irvine, USA), reduction of the septolateral tricuspid annular diameter was a marker of procedural success. *Table 1* summarizes all trials and the employment of imaging indices.

**Table 1:** Summary of the imaging parameters assessed in interventional studies

Parameters	CLASP-TR^40^	PASTE^39^	TRAVEL^36^	TRICUS EURO^38^	TRILUMINATE Pivotal^32^	TRISCEND/TRISCEND II Pivotal^31^	TRI-REPAIR^41^
Tricuspid valve parameters							
PISA related parameters	√	√	√		√	√	√
VC	√	√	√		√	√	√
3D VCA						√	
HVF pattern				√		√	
TV DMG		√				√	
TR jet area	√				√		
Anatomical measurements							
RV dimensions	√	√	√	√	√	√	√
RV volumes						√	
TA dimensions	√			√	√	√	√
RA dimensions	√	√	√	√	√	√	√
IVC dimensions	√		√		√	√	
Functional parameters							
TAPSE	√	√		√	√	√	√
RV FAC	√		√		√	√	√
RV strain				√	√		
PASP		√	√		√	√	√
TAPSE/PASP		√				√	
LVEF	√		√		√	√	√
LVOT SV/CO	√				√	√	

### Focus on tricuspid valve

TVARC^34^ has recommended a comprehensive, multi-parametric approach for the assessment of TR, incorporating both two-dimensional and three-dimensional TTE with a combination of qualitative, semiquantitative, and quantitative parameters.^42^ Cardiac magnetic resonance (CMR) serves as a reasonable alternative imaging modality; however, it is important to recognize that the grading thresholds may differ between echocardiography and CMR due to inherent differences in measurement techniques and volume quantification standards.^43^ For baseline assessment prior to intervention, a full spectrum of qualitative, semiquantitative, and quantitative parameters should be used to ensure comprehensive evaluation. However, following TTVI, the accuracy of certain semiquantitative and quantitative measures may be compromised depending on the specific type of intervention performed. For example, after TEER, the vena contracta (VC) width may misjudge severity in the presence of multiple regurgitant jets, given the absence of validated criteria for summation of multiple VC widths. Similarly, proximal isovelocity surface area (PISA)-derived effective regurgitant orifice area (EROA) and regurgitant volume (RVol) may be prone to overestimation due to distortion of flow convergence geometry following device implantation. Volumetric pulsed-wave Doppler based approach to TR quantification has not been well studied.^44^ In addition, post-procedural hepatic vein flow patterns may not reliably correlate with residual TR severity, as longstanding right atrial remodelling and reduced compliance from chronic TR can alter venous flow independent of regurgitant severity. Furthermore, TR assessment remains highly sensitive to volume status, respiration, and arrhythmias, underscoring the current absence of a universally accepted gold standard for quantification. Among available methods, 3D VC area measurement offers a reasonable approach for both baseline and post-procedural assessment, although it is not without technical limitations and requires further validation. There remains a critical need to develop and refine integrated algorithms capable of reconciling discordant parameters and improving diagnostic accuracy. In addition to regurgitant measurements, continuous-wave Doppler interrogation should include assessment of pressure half-time, transtricuspid diastolic velocity-time integral, peak and mean gradients, and peak and mean TR gradients.^34^

Documentation of device related complications should include device dislodgement, device dysfunction includes residual or recurrent *trans*- or paravalvular leak, leaflet thickening, reduced leaflet motion, leaflet thrombosis, frame fracture (for valve replacement), endocarditis and device erosion. 3-dimensional echocardiography or full-cycle computed tomography scan can be utilized to assess valve area.^34^

TR was universally classified using the 5 grade scheme in studies on TTVI devices in keeping with the TVARC^34^ recommendation. All studies assessed TR with PISA EROA, and most with RVol and mean VC width including TRILUMINATE Pivotal^32^ that studied the TriClip (Abbott Structural Heart, Santa Clara, USA) and TRAVEL^41^ which evaluated the Lux Valve (Jenscare Scientific, Ningbo, China). DMG was assessed in TRISCEND^42^ & PASTE^44^ and HVF in TRICUS EURO^43^ evaluating the TricValve system (P+F Products, Vienna, Austria). Of note, in PASTE,^44^ TR pre-TTVI was quantified with mean VC width and PISA EROA and, post-TTVI, the average width of only the largest jet was assessed.

### Shifting the focus to right and left heart

Although not used as study endpoints, right heart dimensions and function were frequently assessed across studies on TTVI. Most studies include end diastolic RV basal and mid diameters, length, end diastolic and systolic volumes. Additionally, TVARC^34^ suggests measuring RV outflow tract diameter in parasternal long and short axes views, TA in parasternal views, RV wall thickness in subcostal view.

### Impact of haemodynamic parameters on clinical outcomes

The imaging assessment of RV function is load dependent. RV function may appear different when loading condition changes or regional dysfunction is present. Despite these limitations, these parameters were associated with outcome after interventions.^34^

**Figure 1:**
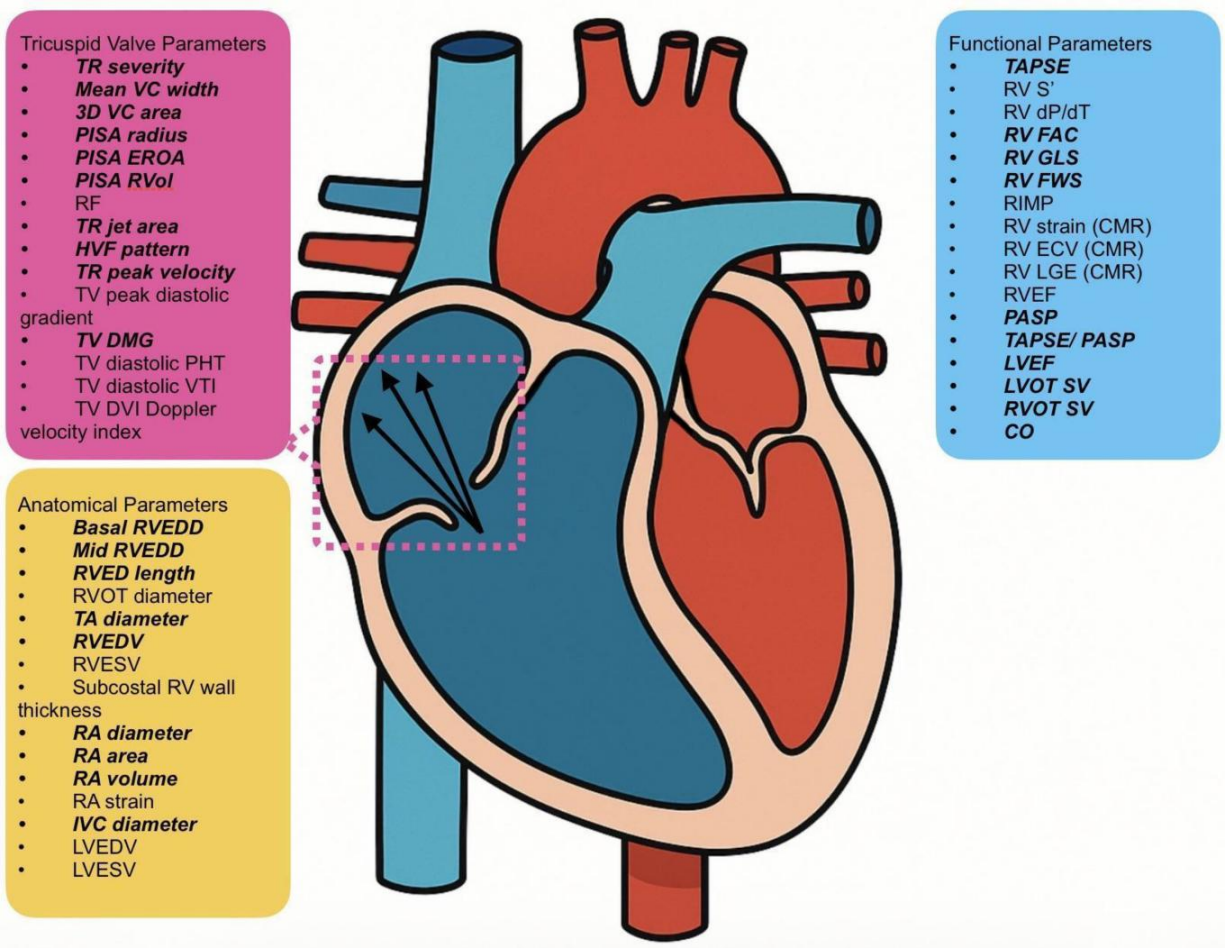
Summary of the imaging parameters recommended by TVARC^4^ with parameters assessed in interventional trials in bold and italic.

Most clinical studies evaluating RV function (*Figure 1*) have traditionally focused on tricuspid annular plane systolic excursion (TAPSE) and RV fractional area change (FAC) due to their relative ease of measurement and established prognostic value. Taking into consideration their limitations such as reproducibility and load dependency, selection of the specific echocardiographic indices as surrogates for RV function, is highly suboptimal. TVARC recommends a more comprehensive assessment that includes tissue Doppler-derived tricuspid systolic velocity (RVS’), global longitudinal strain, free wall strain, RV ejection fraction (EF), and RV–PA coupling. RV–PA coupling reflects the ability of the right ventricle to adapt its contractile function in response to changes in afterload. Effective coupling signifies adequate compensation to maintain cardiac output despite varying load, whereas uncoupling represents a failure of this adaptive mechanism, leading to impaired cardiac output and adverse outcomes. Assessment of pulmonary arterial systolic pressure (PASP) is notoriously problematic in severe TR, especially with increasing severity and a triangular TR continuous wave signal. Moreover, TAPSE is not an optimal surrogate of global RV function as it only measures the longitudinal motion of the RV base and it can both overestimate and underestimate RV performance. There was conflicting evidence on whether baseline TAPSE, FAC, RV end diastolic area and PASP predict clinical outcomes after TTVI.^35,36^ TAPSE and FAC tended to improve in those with pre-existing RV dysfunction and worsen or remain unchange in those with normal baseline RV function. But these changes were not associated with clinical outcomes.^40^ Despite these limitations, the ratio of TAPSE to PASP (TAPSE/PASP) was shown prognostic significance in patients both before and after tricuspid TEER.^37,38^ Invasive PASP assessment provides superior accuracy, but widespread use may be limited by invasiveness and associated risks of right heart catheterization.^39^ Alternative imaging approaches such as cardiac computed tomography or 3-dimensional echocardiography allow for assessment of RV SV indexed to RV end-diastolic volume, offering a non-invasive surrogate for RV function and RV–PA coupling.^45^ However, data supporting the prognostic value of these volumetric indices remain limited, highlighting the need for further research to validate their clinical utility.^34^

Cardiac output should be measured with invasively or noninvasively (echocardiography, inert gas rebreathing spirometry or CMR) as an increase in SV and cardiac output, presumably related to TR reduction, has been noted.^40,41^

The measurements used in TTVI studies are summarized in *Table 1* and *Figure 1*. Of note, TAPSE/PASP was only assessed in TRISCEND II Pivotal^31^ and PASTE.^44^  *Table 2* summarizes the suggested reproducibility, accessibility, sensitivity and prognostic implications of these measurements.

**Table 2:** Table comparing different imaging parameters on reproducibility, accessibility, sensitivity and prognostic values^31,32,34,36,38–41,46–49^

Parameters	Reproducibility		Accessibility		Sensitivity		Prognostic value	
	Pre	Post	Pre	Post	Pre	Post	Pre	Post
Tricuspid valve parameters								
PISA related parameters	+++	+/−	+++	+++	+++	+/−	+++	++
VC	+++	+/−	++++	++++	+++	+/−	+++	++
3D VCA	+++	+++	++	++	++++	++++	++	+
HVF pattern	++++	++++	++++	++++	++	++	++	+
TV DMG	++++	++++	++++	++++	+	+	+	+ for TEER +++ for TTVR
TR jet area	++	+	+++	+++	++	++	++	+
Anatomical measurements								
RV dimensions	++	++	++++	++++	++	++	+/−	?
RV volumes	+++	+++	+	+	+++	+++	++	?
TA dimensions	++	++	++++	++++	++	++	+	?
RA dimensions	++	++	++++	++++	++	++	++	?
IVC dimensions	++++	++++	++++	++++	++	++	+/−	?
Functional parameters								
TAPSE	+++	+++	++++	++++	++	++	++	+/−
RV FAC	++	++	+++	+++	++	++	++	+/−
3D RVEF	++	++	+	+	+++	+++	?	?
RV strain	++	++	++	++	++	++	?	?
PASP	+++	+++	++++	++++	++	+++	+/−	?
TAPSE/PASP	++	++	++++	++++	++	++	++++	+++
LVEF	+++	+++	++++	++++	+	+	++	?
LVOT SV/CO	+++	+++	++++	++++	+	+	++	?

3D, 3 dimensional; CMR, Cardiac magnetic resonance; CO, Cardiac output; CT, Computer tomography; CWD, Continuous wave Doppler; DMG, Diastolic mean gradient; DVI, Doppler velocity index; ECV, Extracellular volume; EDD, End diastolic diameter; EDV, End diastolic volume; EF, Ejection fraction; ESV, End systolic volume; EROA, Effective regurgitant orifice area; FAC, Fractional area change; FWS, Free wall strain; GLS, Global longitudinal strain; HVF, Hepatic vein flow; IVC, Inferio vena cava; LGE, Late gadolinium enhancement; LV, Left ventricle; LVOT, Left ventricular outflow tract; PASP, Pulmonary artery systolic pressure; PHT, Pressure half time; PISA, Proximal isovelocity surface area; PLAX, Parasternal long axis; PR, Pulmonary regurgitation; PSAX, Parasternal short axis; PW, Pulsed wave; RA, Right atrial; RF, Regurgitant fraction; RIMP, Right ventricular index of myocardial performance; RV, Right ventricle; RVol, Regurgitant volume; RVOT, Right ventricular outflow tract; SV, Stroke volume; TA, Tricuspid annulus; TAPSE, Tricuspid annular plane systolic excursion; TDI, Tissue Doppler imaging; TR, Tricuspid regurgitation; TTE, Transthoracic echocardiogram; TV, Tricuspid valve; VC, Vena contracta; VTI, Velocity time integral.

## Comparison between TEER and TTVR

In the TRILUMINATE trial,^32^ the 3-year results show durable reduction in TR and sustained clinical benefit, but they don’t specifically stratify by baseline RV strain. There is a report^46^ that mentions that RV free-wall strain went from ∼ −15.3% at baseline to −6.4% after the procedure (i.e. less negative, worse) in the acute period. Over the mid-term,^47^ reverse remodelling and some recovery of function are observed. Remarkably, a decline in RV function was seen in patients with *normal* RV function at baseline, but not in those who already had RV dysfunction.^50^ However even in the latter manuscript, RV function surrogate was TAPSE which represents the excursion of the RV free wall only and it is a volume dependent echo index. Traditionally TAPSE has been overused but does not reflect RV global function and of course it may lead into unreliable conclusions.

In the early clinical experience of EVOQUE, reduced RVEF (≤ 45%) measured by 3D echo has been flagged as an independent predictor of worse outcomes.^48^ In the TRISCEND II pivotal trial,^37^ inclusion criteria excluded patients with ‘severe right ventricular dysfunction’ (defined in the protocol as RVEF < 25% by 3D echocardiography or visually severe impairment). Again, this inclusion criterion is rather subjective as there is a variability between observers when it comes to visual assessment. Data on strain or RV contractile reserve are still lacking and attention should be drawn on optimal RV assessment.

The limited data suggest that better baseline RV function is a favourable prognostic marker, particularly in TTVR with EVOQUE. It is plausible that patients with severe baseline impairment may derive less benefit (or incur more risk) from more ‘aggressive’ interventions (like full valve replacement) compared to a more moderate repair approach. However the lack of a standardized approach on RV assessment indicates that a detailed pre-procedural assessment as per guidelines^49^ would be more optimal for successful procedural outcomes.

### Knowledge gaps

At present, TTVI has not been proven to have a significant impact on hard outcomes such as mortality or heart failure hospitalization although the reduction in TR severity has been associated with improved quality of life. The commonly accepted outcome predictors were mostly noted from observational studies. There is only a short follow up period in the currently available limited number of randomized trials on a limited range of TTVI devices. The ongoing randomized trials may release hints to facilitate patient selection to optimize clinical outcomes.

On the other hand, imaging parameters were not uniformly assessed amongst different trials or studies. Newer imaging techniques such as strain^51^ or 3D echocardiographic parameters^52^ were not systematically assessed in most studies. Given their ability to predict outcomes outside the context of TTVI, the inclusion of these parameters in clinical settings or studies may detect findings that could not be revealed by simpler tools such as TAPSE or FAC. There is also a lack of data on RV contractile reserve with exercise echo.^53^ While technically challenging, requiring a learning curve for the imager who is performing the test, RV contractile reserve and uncoupling has been proven to be the cornerstone of outcome post structural interventions.

### Clinical implications

At the moment, symptom improvement was seen after TTVI regardless of baseline characteristics. Patients may fare worse outcomes if there were significant pre-existing RV dysfunction or RV-PA decoupling. Therefore, until further data is available, clinicians may either offer TTVI before adverse RV remodelling happens or when decision to perform TTVI was made TTVI by the heart team, more intense clinical or imaging follow up, with shorter follow up intervals than that of the clinical trials, should be arranged. However, the absence of proven therapy for RV dysfunction should be highlighted. Imaging protocols both before and after TTVI should include those assessed in clinical trials and recommended by TVARC. On top of that, 3D RV parameters and strain imaging may be performed. But noting the time constraint, reproducibility and accessibility in real world clinical practice, one may opt to leave out certain parameters after TTVI (such as PISA related parameters if the flow convergence zone was significantly distorted or 3D RVEF with suboptimal image quality).

An optimal evaluation of post-procedural outcomes following TTVI should adopt a balanced approach that integrates both imaging and functional endpoints—such as preservation or improvement of RV function and RV–PA coupling and clinical endpoints, including improvements in quality of life and functional status. Comprehensive assessment across this spectrum of outcomes is essential to fully capture the clinical benefit of these interventions. A critical insight from current clinical trials is the recognized need for long-term data to assess the durability and sustained efficacy of these devices. While early improvements in symptomatic and functional parameters are encouraging, the long-term stability of device performance and its impact on RV remodelling, RV–PA coupling, heart failure hospitalizations, and overall survival remain incompletely defined. Importantly, future research should move beyond a valve-centric perspective to a more integrated view that considers both valvular correction and its downstream effects on right ventricular structure and function. This paradigm shift may ultimately yield a more accurate prediction of clinical outcomes, guiding patient selection, procedural planning, and longitudinal management strategies.

### Future perspectives

The ongoing advancement of multimodality imaging holds significant promise for improving patient selection in structural tricuspid interventions, enabling identification of candidates with the greatest potential for favourable long-term outcomes. Assessment of RV performance and contractile reserve is particularly critical prior to intervention, as preserved contractile reserve has been associated with improved postoperative RV adaptation and clinical prognosis. In addition, emerging imaging techniques such as CMR and molecular imaging may allow for early detection of RV fibrosis, which could serve as a marker of adverse remodelling and help identify patients at risk for suboptimal procedural outcomes. Similarly, the assessment of multiorgan involvement, including hepatic extracellular volume^54^ as an early indicator of cardiohepatic syndrome, may facilitate timely intervention before irreversible organ dysfunction occurs, shifting the management paradigm from passive monitoring to proactive treatment. As it is well known that the develop of cardiohepatic and cardiorenal syndromes may prohibit successful outcomes,^55^ early liver impairment detection with imaging is a promising imaging index within the risk score of TR patients.

Accurate volumetric quantification of TR remains a major challenge in the tricuspid space. Advanced imaging modalities such as 3-dimensional echocardiography and CMR offer promise for pre and post-procedural TR quantification. Serial volumetric measurements can provide objective markers of reverse remodelling and functional recovery, offering early signals of procedural success or failure.^56^ Given the complex geometry of the right heart and limitations of two-dimensional imaging, standardized, reproducible volumetric assessment is critical for meaningful interpretation of outcomes and for guiding longitudinal management in this evolving field.^57^

From a clinical perspective, the establishment of dedicated, multidisciplinary valve clinics within tertiary care centres represents another critical step towards improving early detection, comprehensive evaluation, and coordinated management of patients with TR and RV dysfunction. Furthermore, expanding clinical research efforts to include randomized trials in patients with pulmonary hypertension, evaluating the combination of targeted medical therapies and invasive structural interventions, will be essential for fully elucidating the complex pathophysiology of RV failure and optimizing therapeutic strategies.

## Conclusion

In conclusion, the focus of TR management should evolve from simply targeting reduction of TR jet and short-term quality of life improvements towards the preservation and restoration of RV myocardial function. Early diagnosis and timely intervention are critical determinants of long-term prognosis, as they may prevent irreversible RV remodelling and functional decline.

## Author contributions

Julia Grapsa; Jonathan Lee (Data curation:Lead; Formal analysis:Lead; Investigation:Lead; Methodology:Lead); Eirini Beneki; Nikolaos Katsanakis; Edoardo Zancanaro; Monica Mukherjee; Edgar Argulian

## Data Availability

No new data were generated or analysed in support of this research

## Lead author biography

Dr. Jonathan Lee is an imaging cardiologist at Queen Mary Hospital, Hong Kong, where he is involved in the Structural Heart Programme. Dr. Lee obtained his medical degree from the University of Liverpool, United Kingdom, in 2013. He completed his specialist training in cardiology at Queen Mary Hospital and furthered his expertise with a fellowship in multimodality cardiovascular imaging at Guy’s and St Thomas’ Hospitals, London, in 2025. His clinical and research interests focus on valvular heart disease and structural heart interventions, with a particular emphasis on the role of advanced imaging in guiding transcatheter therapies.
